# Free-Standing N-Doped Porous Carbon Fiber Membrane Derived From Zn–MOF-74: Synthesis and Application as Anode for Sodium-Ion Battery With an Excellent Performance

**DOI:** 10.3389/fchem.2021.647545

**Published:** 2021-04-16

**Authors:** Kaiwen Xue, Yechen Si, Shuya Xie, Jingxuan Yang, Yan Mo, Baojun Long, Wen Wei, Peiyu Cao, Huixian Wei, Hongyu Guan, Elizabeth G. Michaelis, George Guo, Yanfeng Yue, Changsheng Shan

**Affiliations:** ^1^Collaborative Innovation Center for Advanced Organic Chemical Materials Co-constructed by the Province and Ministry, College of Chemistry and Chemical Engineering, Hubei University, Wuhan, China; ^2^Center for Advanced Analytical Science, c/o School of Chemistry and Chemical Engineering, Guangzhou University, Guangzhou, China; ^3^Department of Chemistry, Northeast Normal University, Changchun, China; ^4^Department of Chemistry, Delaware State University, Dover, DE, United States; ^5^Dover High School, Dover, DE, United States

**Keywords:** carbon fiber, metal-organic framework, sodium ion battery, heteroatom doping, porous structure, electrochemistry

## Abstract

It is important to develop new energy storage and conversion technology to mitigate the energy crisis for the sustainable development of human society. In this study, free-standing porous nitrogen-doped carbon fiber (PN-CF) membranes were obtained from the pyrolysis of Zn–MOF-74/polyacrylonitrile (PAN) composite fibers, which were fabricated *in situ* by an electrospinning technology. The resulting free-standing fibers can be cut into membrane disks and directly used as an anode electrode without the addition of any binder or additive. The PN-CFs showed great reversible capacities of 210 mAh g^−1^ at a current density of 0.05 A g^−1^ and excellent cyclic stability of 170.5 mAh g^−1^ at a current density of 0.2 A g^−1^ after 600 cycles in sodium ion batteries (SIBs). The improved electrochemical performance of PN-CFs can be attributed to the rich porous structure derived by the incorporation of Zn–MOF-74 and nitrogen doping to promote sodium ion transportation.

## Introduction

In recent years, sources of clean energy, such as solar and wind energy, have developed rapidly due to the energy crisis, environmental pollution, and greenhouse effect from fossil fuels (Green et al., 2017; Boretti, 2020; Ghenai et al., 2020; Gorjian et al., 2020; Huang et al., 2020; Mohammadnia et al., 2020). However, it is challenging to integrate intermittent renewable energy into a grid and constantly coordinate the generation and consumption of electricity. Secondary electrochemical batteries are an important device for large-scale energy storage of sporadic electric energy because of their low cost, high-energy conversion efficiency, and convenience (Huang et al., 2019; Deng et al., 2020; Dong et al., 2020; Fang Y. et al., 2020; Lim et al., 2020; Liu H. et al., 2020; Luo et al., 2020; Zhang N. et al., 2020). Lithium-ion batteries (LIBs) are ideal and successful secondary electrochemical batteries for energy storage and conversion. Unfortunately, scarcity (20 ppm) of lithium on the crust of the Earth restricts its large-scale application in energy storage (Liu et al., 2017).

Because of abundance in raw materials, low cost, and electrochemical behavior similar to that of lithium (Kim et al., 2012; Bian et al., 2020; Shen et al., 2020), sodium ion batteries (SIBs) are considered as a promising type of secondary electrochemical battery for large-scale energy storage (Liu et al., 2019). However, compared with lithium ion, sodium ion has a larger diameter, which limits the rate of sodium ion embedding in a host material. This results in a low cycle performance and poor stability of SIBs. Development of advanced materials is a key to realizing the practical application of SIBs. Up until now, the anode materials for SIBs mainly include alloys (Jena et al., 2020; Zheng et al., 2020), metal phosphide (Cui et al., 2016; Fang K. et al., 2020), metal oxides (Jian et al., 2014; Zhao et al., 2015), metal sulfides (Chen et al., 2020; Huang et al., 2020; Zhao W. et al., 2020), and carbon materials (Flores et al., 2018; Kumaresan et al., 2020; Shan et al., 2020). Among them, carbon materials are one of the more promising materials for SIBs because of their excellent electrical conductivity, chemical stability, high energy density, and low cost (Hassan et al., 2020; Samantaray et al., 2020; Sánchez-Romate et al., 2020; Zhang et al., 2020). Graphite is the most successful carbon material in the study of lithium-ion batteries, but, as mentioned above, the diameter of Na^+^ is large, so it is difficult to embed/detach from graphite. This will affect the reversible cycle performance of SIBs and will result in low capacity.

Ge and Fouletier studied the electrochemical intercalation behavior of sodium in graphite in PEO-based electrolytes with a reversible capacity of as low as 35 mAh g^−1^ (Ge and Fouletier, 1988). Later, it was found that hard carbon had a disordered structure and wide interlayer spacing, which is beneficial to the storage of sodium. Stevens and Dahn used hard carbon with SIBs and found that the reversible capacity of SIBs went up to 300 mAh g^−1^ (Stevens and Dahn, 2000). However, a large volume expansion of the hard carbon anode led to the structure being pulverized and unstable (Bai et al., 2017). Carbon fibers are also ideal anode materials for SIBs with their great mechanical strength and high conductivity. Carbon fiber membranes can be fabricated easily by an electrospinning technology (Chee et al., 2020; Lan et al., 2020; Yue et al., 2020; Zhao J. et al., 2020). The resulting carbon fiber membranes have a high specific surface area and good mechanical strength and that could be used as free-standing electrodes without the addition of any binders or additives (Hu et al., 2020). For example, Flores et al. incorporated carbon fibers into SIBs, which showed 330 mAhg^−1^ for their first cycle discharge capacity but only 88 mAh g^−1^ remained after 100 cycles (Flores et al., 2018). Therefore, the performance of carbon-based anodes for SIBs still needs to be improved to meet practical requirements.

Metal organic frameworks (MOFs) are crystal metal–organic hybrid compounds composed of metal ions and organic ligands as nodes and pillars, respectively. These materials have advantages, such as high specific surface area, tunable structure, and composition, and uniform pore size (Liu Q. et al., 2020; Meza-Pardo et al., 2020; Zhang H. et al., 2020). MOFs have been widely used in catalysis, gas separation, energy storage, and other fields (Zou et al., 2018). Due to the instability and poor conductivity of pure MOFs in organic electrolytes, direct application of pure MOFs in battery anodes is rare (Zhang et al., 2018; Gao et al., 2019). Most MOFs are converted into porous carbon-based materials by pyrolysis to improve electrical conductivity and structural stability. Among the MOFs, MOF-74 is a classic microporous material, in which 2, 5-dihydroxy terephthalic acid can coordinate with various metal ions (Glover et al., 2011). After the pyrolysis process, the MOF-74-derived porous carbon has a high specific area, porosity, and conductivity. It can also produce a large amount of gas and huge pore size, which are conducive to the charging/discharging of Na^+^ (Gong et al., 2020).

In this study, we successfully fabricated Zn–MOF-74/PAN composite fibers *in situ* directly from metal salt, organic ligand, and PAN by an electrospinning technology. After the pyrolysis process, Zn–MOF-74/PAN fibers were carbonized into nitrogen-doped carbon fibers with a porous structure. Then, the sodium ion storage properties of the PN-CFs, as anode materials, were studied in SIBs. Results show that the PN-CFs showed obvious enhancement of the SIBs performance compared with N-CFs.

## Experimental Section

### Materials

PAN Mw = 70,000) was purchased from Toray Resin Company (Tokyo, Japan). Methanol, 2, 5-dihydroxyterephthalic acid, zinc acetate, dimethylformamide (DMF), and hydrochloric acid were obtained from Beijing Chemical Works (Beijing, China) and used without further purification.

### Synthesis of Zn–MOF-74/PAN Fibers

Zn–MOF-74/PAN fiber membranes were obtained using an electrospinning technology. First, PAN (3 g) was dissolved in 17 mL N–DMF to obtain a uniform 15 wt% PAN/DMF solution. A 2, 5-dihydroxyterephthalic acid (0.1 g dissolved in 3 mL DMF) solution was added dropwise into the prepared 15 wt% PAN/DMF solution under rapid magnetic stirring. Then, a zinc acetate (0.17 g dissolved in 2 mL DMF) solution was added dropwise to fabricate Zn–MOF-74 *in situ* with PAN. After stirring for 18 h, the uniform Zn–MOF-74/PAN solution was decanted into a 5-mL syringe with a metallic needle of 0.8 mm. A high-voltage DC power supply device (model DW-P303-1ACF0) for electrospinning was bought from Dongwen High Voltage Power Supply Co. Ltd. (Tianjin, China). The distance of the needle from the aluminum foil was 20 cm with a voltage of 10 kV and a flow rate of 0.2 ml/h for the solution, applied by the electrospinning equipment. A soft aluminum was supported on a thick iron plate for collecting of fibers. After electrospinning, the resulting Zn–MOF-74/PAN fiber membranes were obtained by removing them from the aluminum foils.

### Synthesis of PN-CFs

The Zn–MOF-74/PAN fiber membranes were pre-oxidized in a muffle furnace at 240°C for 2 h with a heating rate of 1°C/min. Then, the pre-oxidized fiber membranes were carbonized in a tube furnace at high temperature (500–900°C) for 1 h with a heating rate of 5°C/min in a high-purity argon atmosphere (purity: 99.999%). After cooling naturally in the furnace, the PN-CFs membranes were soaked in a 10% HCl solution for 3 h three times to remove residual Zn. Finally, the fiber membranes were washed with distilled water three times and dried in an oven at 80°C to obtain free-standing porous nitrogen-doped carbon fiber membranes, named PN-CFs-T (T represents carbonization temperature). As a control, the PAN fibers (PAN-NFs) without Zn–MOF-74 and carbonized nitrogen-doped carbon fibers (N-CFs-T) at different carbonization temperatures were fabricated using the same process without the addition of zinc acetate and 2, 5-dihydroxyterephthalic acid.

### Characterization

The PAN fibers, Zn–MOF-74/PAN composite fibers, and final carbonized fibers were identified using a Rigaku SmartLab X-ray diffractometer (XRD, Rigaku Smartlab Beijing Co., Beijing, China) with Cu-K radiation (λ = 1.5418 Å). A scanning electron microscope (SEM, HITACHI SU8000, Hitachi Ltd., Tokyo, Japan) and a high-resolution transmission electron microscope (HRTEM, JEOL-2100F, Japan) were used to characterize the microstructure and surface morphology of the samples. Raman spectroscopy (Ar^+^ laser, λ = 532 nm) was performed to analyze the degree of graphitization of the samples. Elemental components and surface structure of the carbon fibers were analyzed by X-ray photoelectron spectroscopy (XPS, SPECS Phoibos 150MCD, Phoibos, Berlin, Germany). The specific surface area and pore size distribution of PN-CFs and N-CFs were determined by the nitrogen adsorption-desorption technique (ASAP 2020, Micromeritics, United States).

### Electrochemical Measurements

Free-standing PN-CFs-T and N-CFs-T membranes were used as anode electrodes with 1-cm diameter and 0.5–0.8 mg mass loading. Coin-type half cells (CR2030) were made in an Ar-filled glove box (H_2_O, O_2_ < 0.1 ppm, Mbraun Inc., Germany). The electrolytes were 1 M sodium perchlorate in EC/PC/FEC or 1 M sodium trifluoromethyl sulfonate in diethylene glycol dimethyl ether. Metallic sodium was used as a counter electrode. Galvanostatic charging/discharging measurements were conducted under a voltage range of 001–2.5 V using a battery-testing system (LAND CT2001A Instruments, Wuhan Land Electronics Co. Ltd., Wuhan, China). Cyclic voltammetry (CV) was performed using VersaSTAT 3 (Princeton Applied Research, Princeton, NJ, USA). Electrochemical impedance spectroscopy (EIS) was performed using VersaSTAT 3 with a frequency ranging from 10^6^ to 0.1 Hz.

## Results and Discussion

### Characterization and Structure of N-CFs and PN-CFs

Zn–MOF-74/PAN-derived PN-CFs were obtained by electrospinning and carbonization processes, as shown in Supplementary Scheme 1. First, Zn–MOF-74 was synthesized *in situ* with PAN to obtain uniform Zn–MOF-74/PAN composite fibers. After pre-oxidation and carbonization, PN-CFs with a porous structure were obtained. The structure originated from the porous structure of Zn–MOF-74 and the evaporation of Zn with a low boiling point.

**Figure 1:**
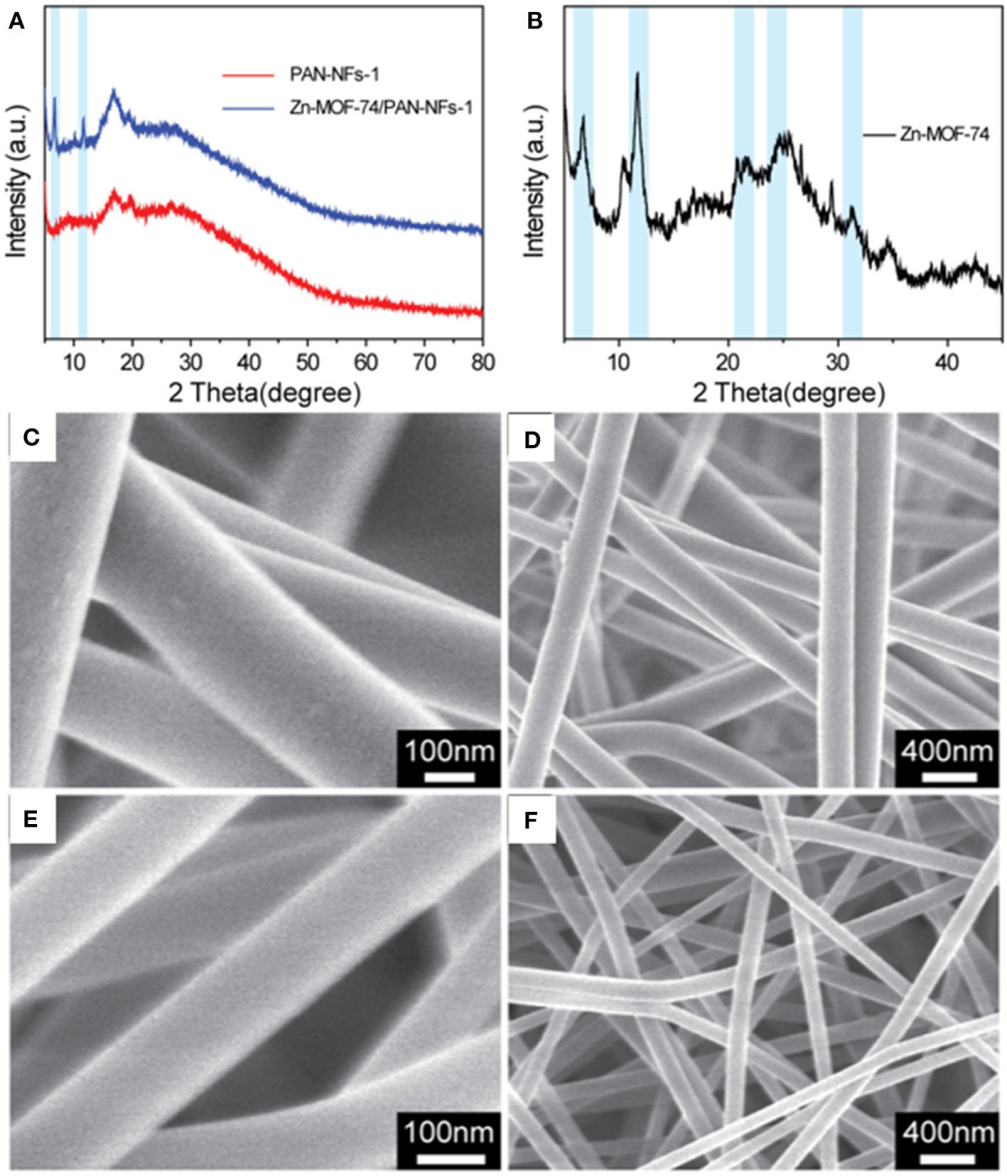
**(A)** XRD patterns of PAN fibers and Zn–MOF-74/PAN composite fibers; **(B)** XRD patterns of Zn–MOF-74 obtained from dissolving Zn–MOF-74/PAN fibers using DMF; **(C,D)** SEM images of PAN fibers; **(E,F)** SEM images of Zn–MOF-74/PAN fibers.

XRD and SEM were performed to verify the successful synthesis of Zn–MOF-74/PAN fibers. In Figure 1A, the XRD curve of the PAN fibers shows both a crystal region and an amorphous region, which is a two-phase quasi-crystal structure. The two weak diffraction peaks at 2θ = 17° and 2θ = 29° (very weak) correspond to the 100 and 110 crystal planes of the PAN fibers (Allen et al., 1994; Ouyang et al., 2015). Compared with the XRD curve of PAN-NFs, the XRD curve of Zn–MOF-74/PAN-NFs shows two new diffraction peaks at 2θ = 6.5° and 11.5°, which correspond to the diffraction peaks of Zn–MOF-74 (the light blue region in Figure 1A) (Caskey et al., 2008). In order to further confirm the presence of Zn–MOF-74 in Zn–MOF-74/PAN-NFs, the PAN in Zn–MOF-74/PAN fibers was dissolved with DMF to remove it, and then the solid residue was investigated by XRD. The XRD curve of the solid residue not only showed the two strong diffraction peaks at 2θ = 6.5 and 11.5 of Zn–MOF-74 but also three other weak diffraction peaks (the light blue region in Figure 1B), which correspond to the diffraction peaks of Zn–MOF-74. These results indicate that the uniform blending of Zn–MOF-74 into the PAN fibers is successful.

In addition, the morphology of the PAN fibers and the Zn–MOF-74/PAN fibers were studied further by SEM. As shown in Figures 1C–F, both the PAN fibers and the Zn–MOF-74/PAN fibers showed a smooth surface, high aspect ratio, and relatively uniform diameter. The diameters of PAN-NFs and Zn–MOF-74/PAN-NFs were about 240 and 140 nm, respectively. In addition, the fibers were interlaced with each other to form a 3D fiber network, which made the material free-stand macroscopically.

**Figure 2:**
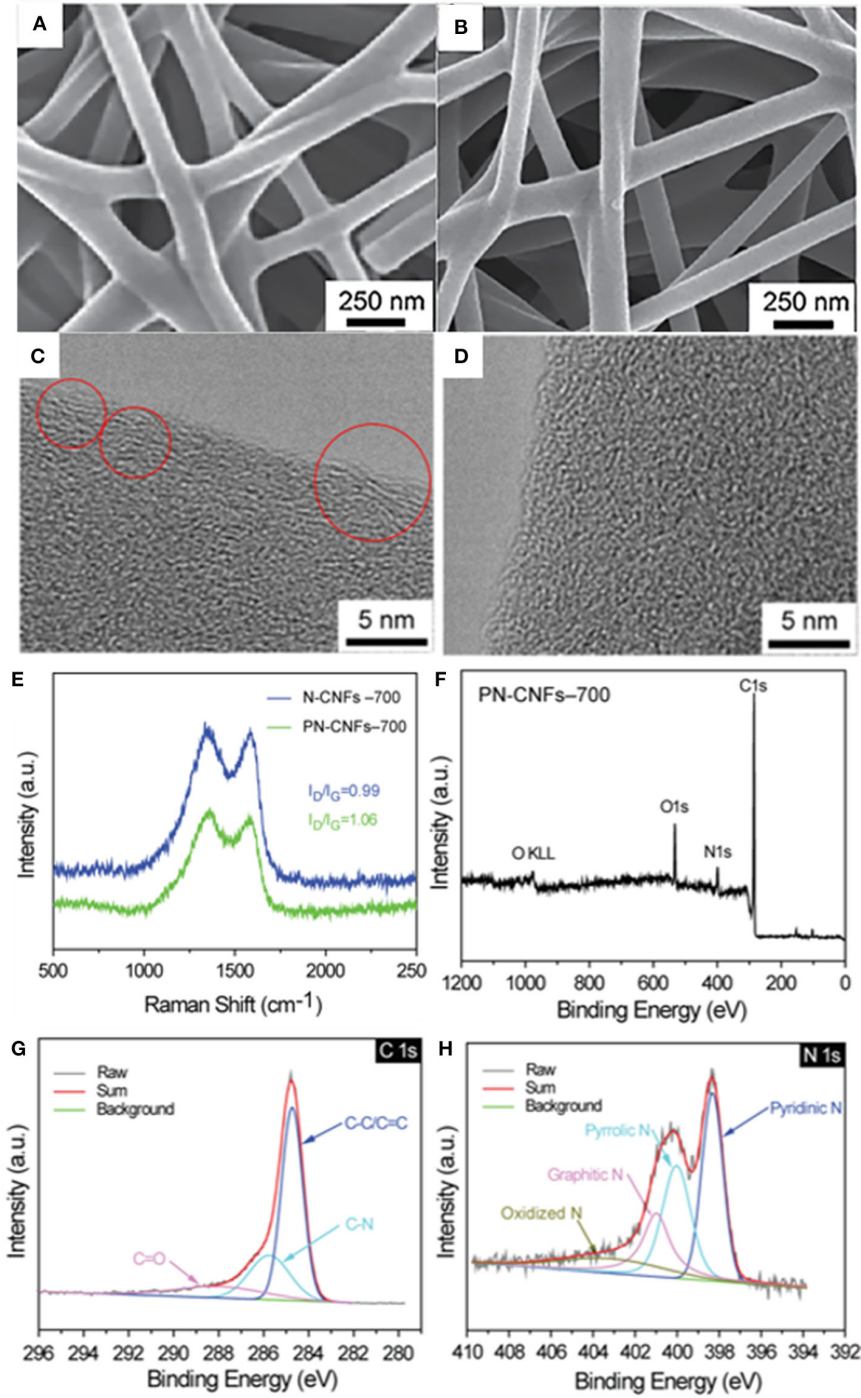
SEM image of N–CFs-700 **(A)** and PN-CFs-700 **(B)**; HRTEM image of PN-CFs-700 **(C)** and N-CFs-700 **(D)**; Raman spectra of N-CFs-700 and PN-CFs-700 **(E)**. The XPS survey **(F)** and high resolution C1s **(G)** and N1S **(H)** spectra of PN-CFs-700.

Then, the PAN fibers and Zn–MOF-74/PAN fibers were carbonized into N-CFs-T and PN-CFs-T by pyrolysis at different temperatures. In Supplementary Figure 1, the XRD patterns of N-CFs-T and PN-CFs-T showed wide peaks at 2θ ≈ 23, which corresponded to the 002 crystal plane of hard carbon materials (Guo et al., 2019), indicating successful fabrication of CFs. The corresponding SEM images of N-CFs-T and PN-CFs-T were characterized and shown in Figure 2 and Supplementary Figures 2–5. Compared with the SEM images of PAN fibers and Zn–MOF74/PAN fibers, the carbonized N-CFs-T and PN-CFs-T still maintained the morphology of the fibers, and the fibers also were connected to each other to form a 3D carbon fiber network. In addition, it can be seen that the diameter of the fiber decreases slightly with a increase in carbonization temperature.

HRTEM was performed to further study the morphology of N-CFs-700 and PN-CFs-700. Both HRTEM images of N-CFs-700 (Figure 2C) and PN-CFs-700 (Figure 2D) showed a disordered structure containing a small amount of graphite microcrystal lattice. This belongs to the typical lattice characteristics of hard carbon materials. N-CFs-700 exhibited more graphitized lattice characteristics (red circle region in Figure 2C) than PN-CF-700, while PN-CFs-700 showed a more disordered porous structure than N-CFs-700. The less graphitized lattice characteristics and obvious porous structure of PN-CFs-700 could be attributed to the presence of Zn–MOF-74.

The graphitization degree of N-CFs-700 and PN-CFs-700 was further characterized by Raman spectroscopy. As shown in Figure 2E, the two broad peaks at 1,360 and 1,600 cm^−1^ in Raman spectra correspond to the D-band and G-band of hard carbon materials, which represent the lattice defect and graphitization structure in carbon materials, respectively. The intensity ratio of D-band to G-band (I_D_/I_G_) represents the disorder degree of carbon materials (Ding et al., 2013). The I_D_/I_G_ value of PN-CFs-700 is 1.06, which is greater than that of N-CFs-700 (0.99). This indicates that PN-CFs-700 have a higher disordered degree than N-CFs-700, which is consistent with the HRTEM results. The higher disordered carbon structure could possibly be attributed to the presence of Zn–MOF-74, which caused more defects in PN-CFs during pyrolysis.

The specific surface area and pore size distribution of PN-CFs and N-CFs were determined by the nitrogen adsorption–desorption technique. The nitrogen adsorption-desorption isotherms and the pore size distributions of the PN-CFs and the N-CFs are shown in Supplementary Figure 6. The Brunauer–Emmett–Teller (BET) surface area of PN-CFs was 2.6 m^2^/g, which was a little higher than that of the N-CFs (2.0 m^2^/g). The PN-CFs showed two main pore-size peaks at 2.2 and 4.2 nm, while the N-CFs only showed one peak at 2.0 nm. The increase in surface area and the new pore structure formation of PN-CFs could be attributed to the addition of ordered porous Zn–MOF-74, in which the organic ligands were decomposed into small gaseous molecules. The improved surface area and the new pore formation of PN-CFs would further facilitate the diffusion of Na^+^ and enhance electrochemical performance.

XPS was performed to characterize the element composition and bonding between the elements on the surface of the materials. XPS survey spectra in Figure 2F and Supplementary Figure 7a showed the presence of C, N, and O elements in PN-CFs-700 and N-CFs-700. In Figure 2G, the C1s spectrum can be resolved into three different peaks at 284.7, 285.8, and 288.5 eV, which are assigned to the bonding of C–C, C–N, and C=O, respectively. In the N1s spectrum (Figure 2H), the four peaks at 398.4, 400.1, 401.1, and 403.1 eV correspond to pyridinic-N, pyrrolic-N, graphitic-N, and oxidized-N, respectively. XPS results indicate that N atoms were successfully doped into carbon fibers (Wang et al., 2018).

In addition, energy dispersive spectroscopy (EDS) mapping was also performed to characterize the element composition and distribution of N-CFs-700 and PN-CFs-700. As shown in Supplementary Figures 8, 9, the SEM mapping of N-CFs-700 and PN-CFs-700 showed the presence of C, N, and O with uniform distribution in the fibers, thus indicating that both N-CFs-700 and PN-CFs-700 are nitrogen-doped carbon fibers.

### Sodium Storage Performance of N-CFs-T and PN-CFs-T

The electrolyte effects of carbonates (EC/PC/FEC, 1 M sodium perchlorate) and ethers (diethylene glycol dimethyl ether, 1 M sodium trifluoromethyl sulfonate) on the electrochemical performance of PN-CFs-700 and N-CFs-700 in SIBs were investigated. Supplementary Figure 10 shows the comparison of the rate performance of N-CFs-700 and PN-CFs-700 in carbonate electrolytes and ether electrolytes. The capacities of the PN-CNTs-700 in ether electrolyte were higher than those in carbonate electrolyte, especially at a high current density. In addition, the ether electrolyte significantly improved the first-cycle Coulomb efficiency of both materials in SIBs. The improvement in capacity and Coulomb efficiency was attributed to the formation of a thinner, more stable, and denser solid electrolyte interface (SEI) membrane on the surface of the material in ether electrolyte (Han et al., 2017). Therefore, all the subsequent electrochemical experiments were tested with ether electrolyte.

The effect of carbonization temperature on SIB performance was also investigated in detail. Supplementary Figure 11 shows the comparison of N-CNFs-T and PN-CNFs-T obtained at different carbonization temperatures ranging from 650 to 900°C. It can be seen that the best carbonization temperature was determined to be 700°C, at which N-CNFs-700 and PN-CNFs-700 exhibited the best capacity performance.

CV was an effective method to study the electrochemical charging and discharging behavior of PN-CF-700 and N-CFs-700 in SIBs. The relationship between peak current and scanning rate follows formulas (1) and (2); where i is the peak current, v is the scanning rate, and a and b are constants related to the electrochemical reaction mechanism, while the value of b is the slope of formula (2). When b = 1, the reaction is surface-controlled (such as adsorption), which belongs to a pseudo-capacitive process; when b = 0.5, the reaction is diffusion-controlled (such as an intercalation process) (Qiu et al., 2017).

(1) $$\begin{array}{l} \text{i}=v^{b}a \end{array}$$

(2) $$\begin{array}{l} \log\left(i\right)=b\times \log\left(\;\upsilon \;\right)+\log\left(a\right) \end{array}$$

**Figure 3:**
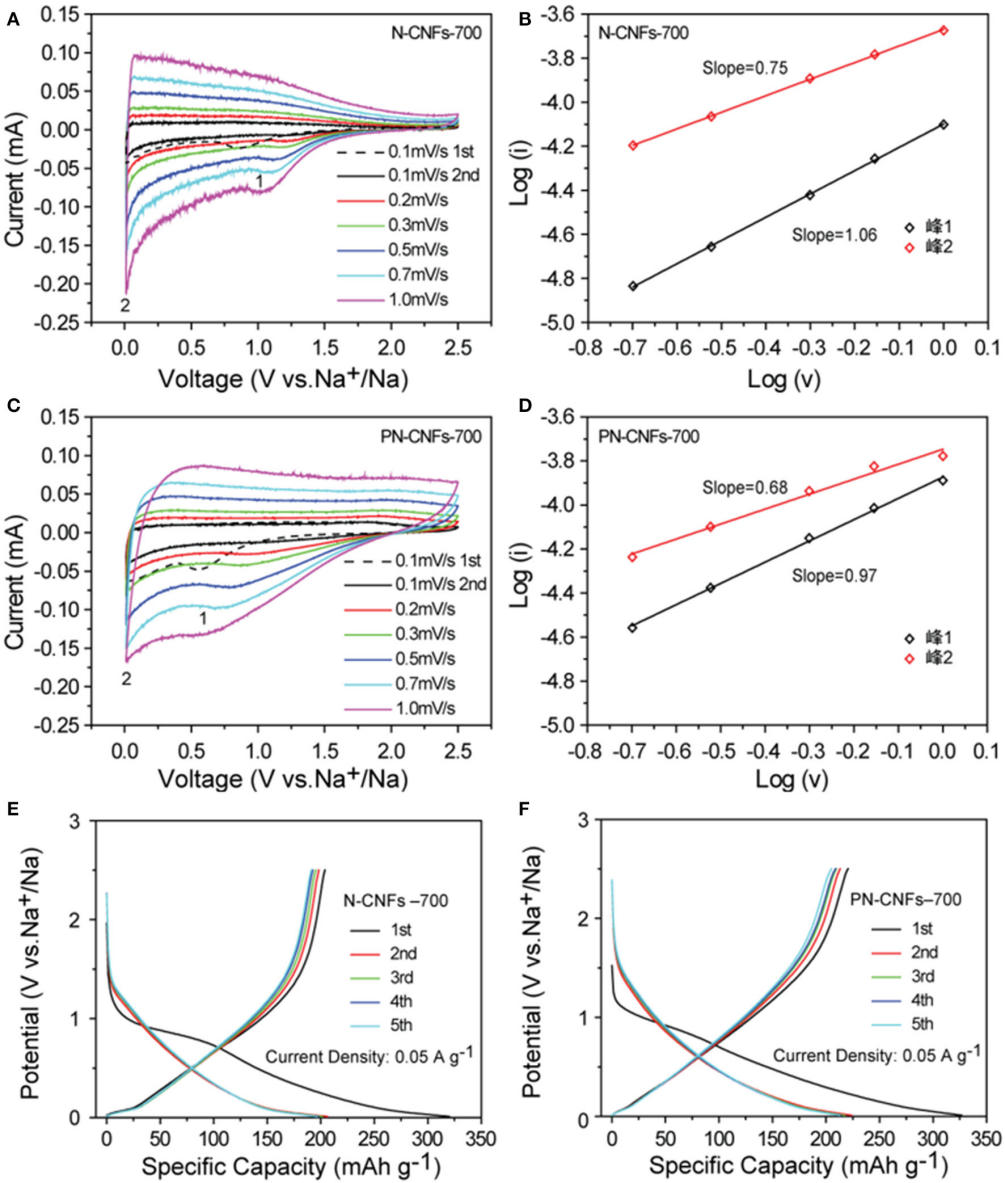
The CV curves of N-CFs-700 **(A)** and PN-CFs-700 **(C)** at 0.01 V−2.5 V (vs. Na^+^/Na) at different scanning rates; The log (i)–log (v) curves of N-CFs-700 **(B)** and PN-CFs-700 **(D)**. The initial five cycles of galvanostatic charge-discharge curves of N-CFs-700 **(E)** and PN-CFs-700 **(F)** at current density of 0.05 A g^−1^ with potential range from 0.01 to 2.5 V (vs. Na^+^/Na).

Figure 3 shows the CV curves of N-CFs-700 and PN-CFs-700 in the voltage range of 0.01–2.5 V with different scanning rates. The CV curves of both N-CFs-700 and PN-CFs-700 showed two similar pairs of redox peaks, indicating two different electrochemical processes in the charging/discharging process. The irreversible wide peaks of about 0.6–0.8 V (the black dotted curves in Figures 3A,C) in the first cycle should be attributed to the decomposition of the solvent in the electrolyte and the formation of SEI membrane. The peaks at 0.6–0.8 V disappeared in the subsequent cycle process, indicating the formation of stable SEI layers. The stable SEI layers can effectively separate the electrode from the electrolyte, thus inhibiting further decomposition of the electrolyte. Figures 3B,D show the linear relationship of log (i)–log (v). The values of b for peaks 1 and 2 in the CV curves of N-CFs-700 were 1.06 and 0.75, respectively, while the PN-CFs-700 showed similar *b* values of 0.97 for peak 1 and 0.68 for peak 2. These results showed that the peak 1 b values were caused by the pseudo-capacitance contribution of the doped nitrogen atoms and the pore structure in the carbon fibers, while the peak 2 b values below 0.1 V were caused by the intercalation and detachment of Na^+^ in the carbon fibers. Although the anodic peak of the CV curve of N-CFs-700 in the first cycle was higher than that of PN-CFs-700, the first discharge capacity of N-CFs-700 in the first cycle was lower than that of PN-CFs-700. This is due to PN-CFs-700 having higher pseudo-capacitance contribution as a result of higher specific surface area and the porous structure in PN-CFs-700.

Figures 3E,F show the charging/discharging curves of N-CFs-700 and PN-CFs-700 at 0.05 A g^−1^. The charging/discharging curves of the two carbon fibers can be divided into two main zones: the slope zone above 0.1 V and the platform zone below 0.1 V. The slope zone is related to the pseudo-capacitance behavior caused by the pore structure and defects of the doped nitrogen atoms in carbon fibers, while the platform region is caused by the intercalation and detachment of Na^+^ in carbon fibers. It can be seen that the capacity of carbon fibers was mainly contributed by the long slope zone, while the short platform zone contributed less to the capacity compared with the slope zone. Therefore, the sodium ion storage capacity of N-CFs-700 and PN-CFs-700 mainly comes from the pseudo-capacitance contribution. The improvement in PN-CFs-700 capacity was mainly due to the enhancement of pseudo-capacitance caused by the porous structure and nitrogen doping of carbon. It was noted that the Coulombic efficiencies of PN-CFs-700 and N-CFs-700 were low in the first cycle. On one hand, the low Coulombic efficiency in the first cycle was presumably related to a kinetic hindrance in intercalation of Li ions into CFs. This required a few discharge/charge cycles to make full intercalation of Li ions into CFs for maximum Coulombic efficiency (Rothermel et al., 2014). On the other hand, the low Coulombic efficiency in the first cycle could be attributed to the formation of SEI film due to irreversible reaction. After the first cycle, the charging/discharging curve coincided well, demonstrating good cyclic stability and reversibility of N-CFs-700 and PN-CFs-700.

**Figure 4:**
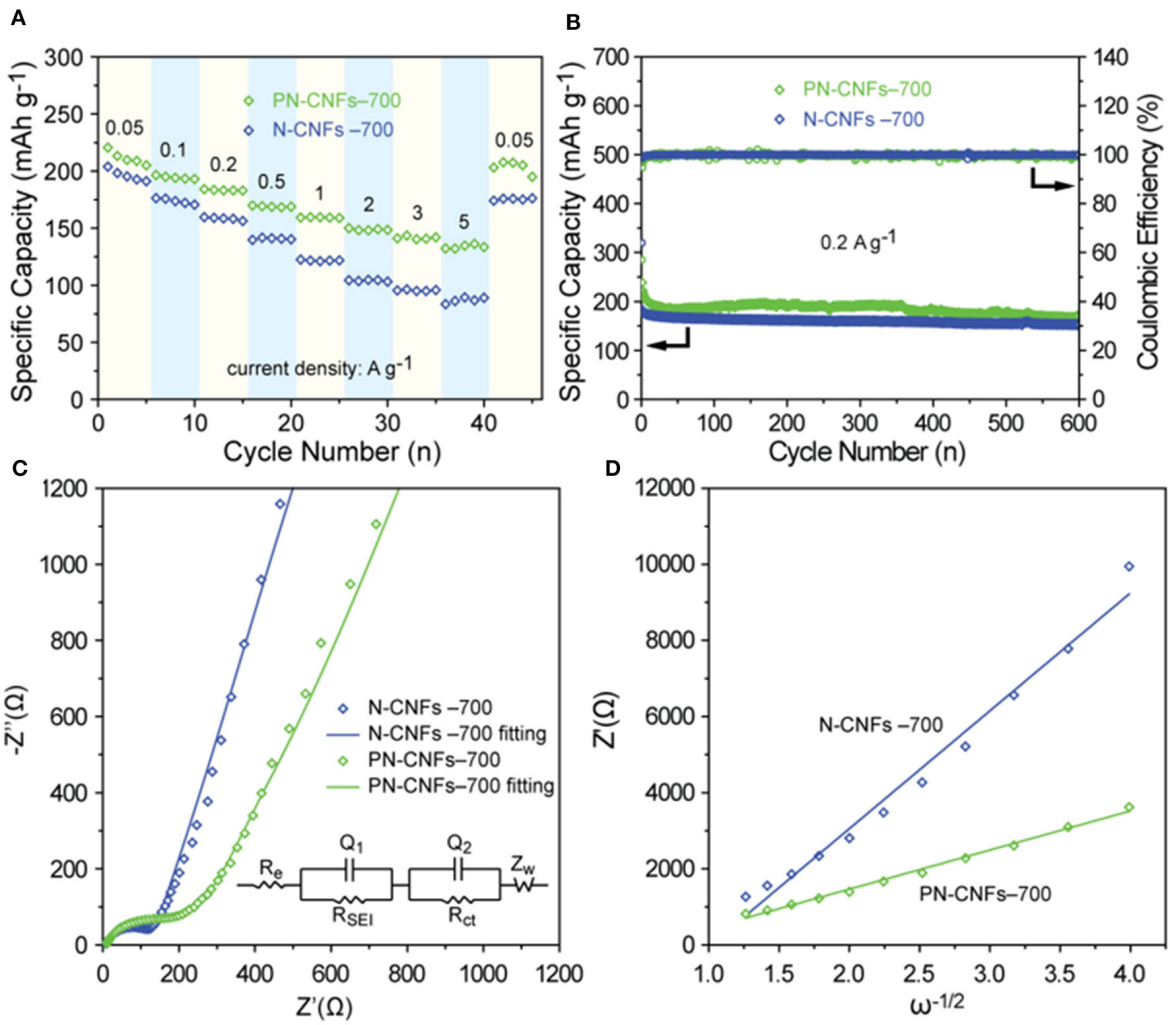
**(A)** The rate performance of N-CFs-700 and PN-CFs-700; **(B)** cycle performance and Coulombic efficiency of N-CFs-700 and PN-CFs-700 for 600 cycles at a current density of 0.2 A g^−1^; **(C)** Nyquist plots of N-CFs-700 and PN-CFs-700 (the inset is the corresponding equivalent circuit diagram); **(D)** Z′ - ω^−1/2^ linear fitting curves in the low frequency zone.

The rate and cycle performance of N-CFs-700 and PN-CFs-700 were also investigated. Figure 4A shows that the capacity of PN-CFs-700 is higher than that of N-CFs-700 at the same current density, especially at high current density. The PN-CFs-700 had a reversible capacity of 210 mAh g^−1^ at 0.05 A g^−1^ and 135 mAh g^−1^ at high current density of 5 A g^−1^. When the current density recovered from 5 to.05 A g^−1^, the reversible capacity could be recovered back to almost the same level as the beginning test. These results showed that the PN-CFs-700 anode had a great rate performance. Figure 4B shows that PN-CFs-700 have a reversible capacity of 170.5 mAh g^−1^ with a capacity retention of 71.4% at 0.2 A g^−1^ after 600 cycles, which is higher than that of N-CF-700. This indicates that PN-CF-700 has excellent cycle stability.

In order to better understand the difference in sodium storage performance between N-CFs-700 and PN-CFs-700, electrochemical impedance spectroscopy (EIS) was performed to analyze the kinetic characteristics of N-CFs-700 and PN-CFs-700 in the electrochemical process. Figure 4C shows the Nyquist plots of N-CFs-700 and PN-CFs-700, and the fitting circuit. The Nyquist plots consisted of the semicircular part at the high and middle frequencies and the linear part at the low frequency. The semicircle diameter represents the charge transfer resistance (R_ct_), and the linear part can be used to evaluate the apparent diffusion coefficient (D_Na_) of Na^+^ in the electrochemical process. In the equivalent circuit diagram, R_e_, R_SEI_, and R_ct_ represent the resistance of the electrolyte, the resistance of the SEI layer, and the charge transfer resistance, respectively. Z_w_ is the Warburg impedance related to the diffusion of Na^+^. The R_ct_ of PN-CFs-700 (170.7 Ω) is greater than that of N-CFs-700 (128.6 Ω). This indicates that the addition of Zn–MOF-74 in PN-CFs-T led to the formation of the porous structure but resulted in lower conductivity of PN-CFs-T due to the disordered graphitic structure in PN-CFs, which has been proved by HRTEM and Raman spectra. In addition, formulas (3) and (4) are used to analyze the curve at low frequency to compare the apparent diffusion coefficient of Na^+^ in N-CFs-700 and PN-CFs-700 in the electrochemical process.

(3) $$\begin{array}{l} D_{Na}=\frac{R^{2}T^{2}}{2A^{2}n^{4}F^{4}C^{2}\sigma^{2}} \end{array}$$

(4) $$\begin{array}{l} Z^{\prime }=R_{D}+R_{L}+\sigma \omega^{-1/2} \end{array}$$

In formula (3), D_Na_, R, T, A, n, F, C, and σ represent the apparent diffusion coefficient of Na^+^, the gas molar constant, the temperature, the surface area of the electrode, the number of electrons transferred in the redox process, the Faraday constant, the concentration of Na^+^, and the Warburg factor, respectively. The ω in formula (4) represents the angular frequency. In formula (3), it is known that the higher σ is, the lower D_Na_ is. σ is associated with Z′ (the real part of the EIS spectrum) through formula (4), and its numerical value is the slope value of formula (4). Figure 4D shows the Z′-ω^−1/2^ linear fitting curves of N-CFs-700 and PN-CFs-700, in which the σ of N-CFs-700 is larger than that of PN-CFs-700. The diffusion rate of Na^+^ in PN-CFs-700 is higher than that in N-CFs-700 in the electrochemical process. This can be attributed to the more disordered porous structure derived from Zn–MOF-74 in PN-CFs-700 to promote the intercalation of Na^+^ in PN-CFs-700. Therefore, the better sodium storage performance of PN-CFs-700 is mainly due to the presence of the porous structure and N doping. The porous structure increased the diffusion rate of Na^+^ and enhanced the pseudo-capacitance of the carbon fibers. The doping of N into carbon fibers can produce large amounts of pyridinic and pyrrolic N on the surface of carbon, which can provide more active sites for Li ion storage and facilitate the transfer of Li ions and electrons (Zheng et al., 2014). The electrochemical performance of PN-CFs was compared to that in some typically reported studies. As shown in Supplementary Figure 12, the PN-CFs did not exhibit an obvious advantage with the capacity at low current density, but the capacity of PN-CFs decreased less than those of the reported carbon materials with increasing current density. Thus, the PN-CFs showed high capacity at a high current density of 5 A g^−1^, which is higher than or compared to those of the reported works.

## Conclusion

In summary, a series of PN-CFs-T membranes were synthesized from Zn–MOF-74/PAN fiber membranes by an electrospinning method. By introducing Zn–MOF-74 into the PAN fibers *in situ*, PN-CFs-T were produced with a disordered porous structure. This increased the Na^+^ diffusion rate in the carbon fibers. The PN-CFs-700 as anode materials obtained at the optimal carbonization temperature showed a high reversible capacity of 210 mAh g^−1^ at a current density of 0.05 A g^−1^ and 135 mAh g^−1^ at 5 A g^−1^. After cycling at a current density of 0.2 A g^−1^ for 600 cycles, PN-CFs-700 still had a high reversible capacity of 170.5 mAh g^−1^ with a 71.4% capacity retention rate. Thus, the resulting free-standing PN-CFs-T membranes exhibited excellent reversible capacity, rate capacity, and cycle stability as an anode for SIBs. The results showed that the disordered porous structure and nitrogen doping could possibly increase the diffusion rate of Na^+^ in the electrochemical process. This made PN-CFs-T higher capacitance to improve sodium ion storage capacity. Therefore, PN-CFs-T are a promising anode material for application in SIBs.

## Data Availability Statement

The original contributions presented in the study are included in the article/Supplementary Material; further inquiries can be directed to the corresponding author/s.

## Author Contributions

YY, HG, and CS conceived the idea, designed the project, and wrote the draft with KX and YS. KX, YS, and SX synthesized PN-CFs and N-CFs and carried out the electrochemical experiments. YY and HG monitored the syntheses and performed all material characterization. All the authors participated in analyzing the results and finalizing the manuscript.

## Conflict of Interest

The authors declare that the research was conducted in the absence of any commercial or financial relationships that could be construed as a potential conflict of interest.
