# Supplementary material for: Free-Standing N-Doped Porous Carbon Fiber Membrane Derived From Zn–MOF-74: Synthesis and Application as Anode for Sodium-Ion Battery With an Excellent Performance
Source: Front Chem. 2021 Apr 16;9:647545. doi: 10.3389/fchem.2021.647545 (PMC8086192; doi:10.3389/fchem.2021.647545)
Supplement: Supplementary file 1 [file Data_Sheet_1.pdf]

Supplementary Information

Free-standing N-doped porous carbon fiber membrane derived from Zn-MOF-74: Synthesis and application as anode for sodium-ion battery with an excellent performance

Kaiwen Xue,[1#] Yechen Si,[1#] Shuya Xie,[2,3] Jingxuan Yang,[2] Yan Mo,[1] Baojun Long, [1] Wen Wei, [1] Peiyu Cao,[1] Huixian Wei,[2] Hongyu Guan,[2, 3]* Elizabeth G Michaelis,[4] George Guo,[4,5] Yanfeng Yue,[4]* Changsheng Shan[1,2]*

[1]Collaborative Innovation Center for Advanced Organic Chemical Materials Co-constructed by the Province and Ministry, College of Chemistry and Chemical Engineering, Hubei University, Wuhan, Hubei 430062, P. R. China.

[2]Center for Advanced Analytical Science, c/o School of Chemistry and Chemical Engineering, Guangzhou University, Guangzhou, Guangdong 510006, P. R. China

[3]Department of Chemistry, Northeast Normal University, Changchun, Jilin 130024, P. R. China

[4]Department of Chemistry, Delaware State University, Dover, Delaware 19901, United States

[5]Dover High School, 1 Dover High Dr, Dover, DE 19904, United States

[#]K.Xue and Y. Si contributed equally to the work.

***Correspondence:**

csshan@hubu.edu.cn (C. Shan); guanhy@gzhu.edu.cn (H. Guan); yyue@desu.edu (Y. Yue).

Key words: carbon fiber, metal-organic framework, sodium ion battery, heteroatom doping, porous structure, electrochemistry.

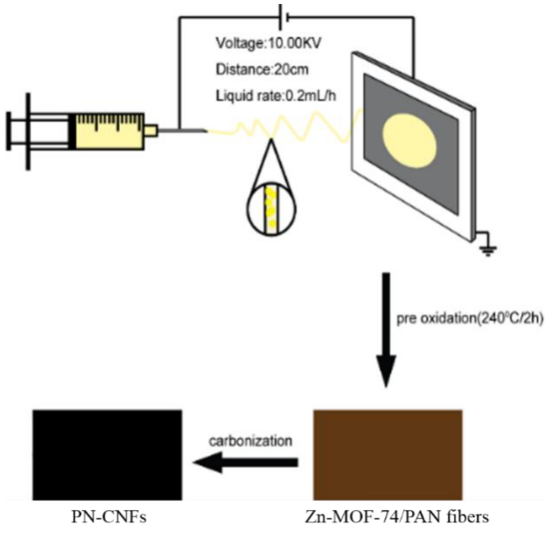

Scheme S1. Schematic diagram of free-standing MOF-74/PAN fibers films by electrospinning technology.

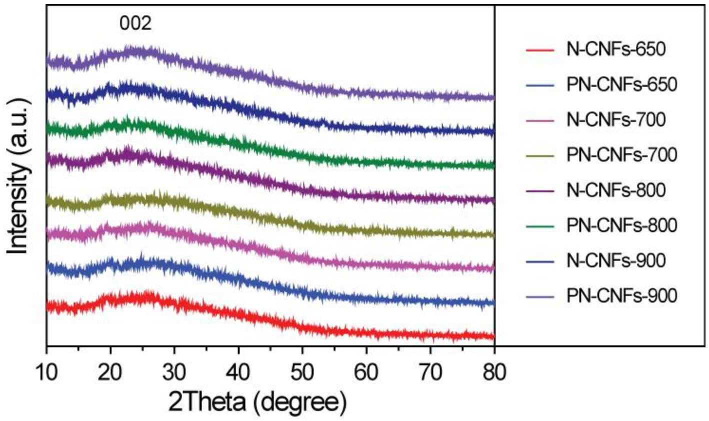

Figure S1. XRD patterns of N-CNFs-T and PN-CNFs-T at different pyrolysis temperature.

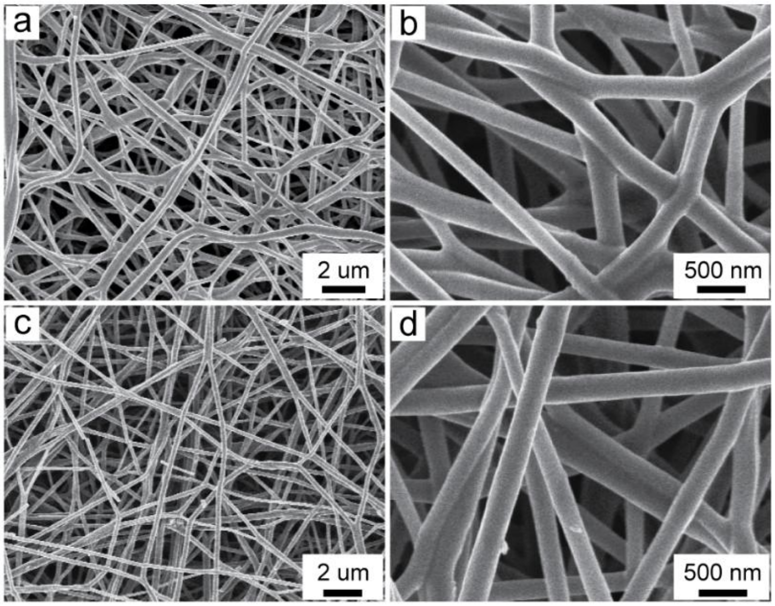

Figure S2. The SEM images of N-CNFs –650 (a, b) and PN-CNFs–650 (c, d)

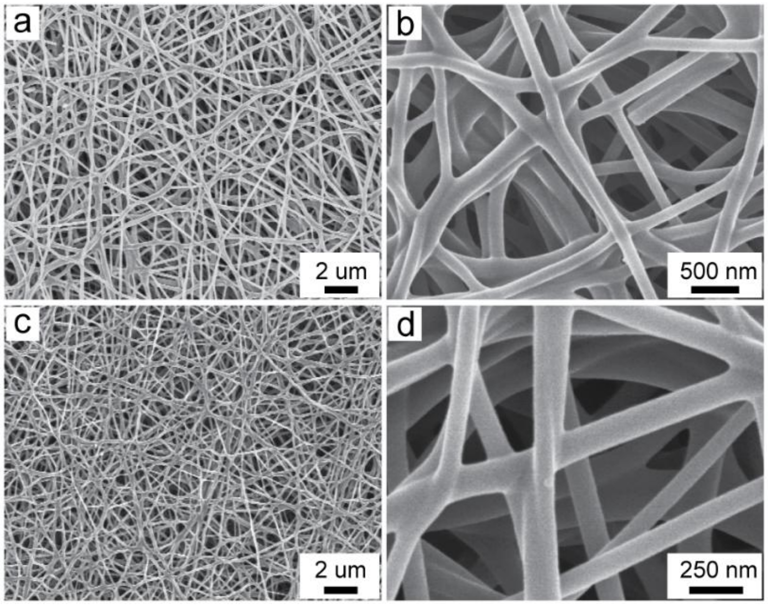

Figure S3. The SEM images of N-CNFs –700 (a, b) and PN-CNFs–700 (c, d)

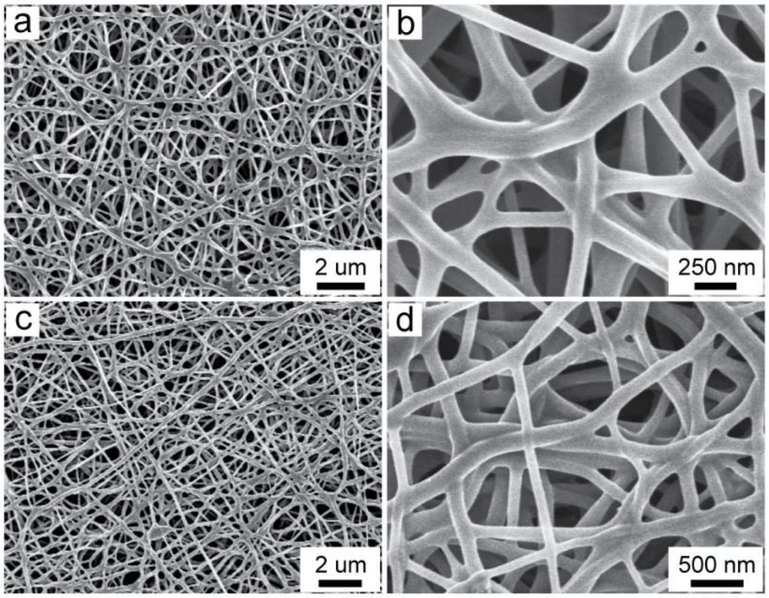

Figure S4. The SEM images of N-CNFs –800 (a, b) and PN-CNFs–800 (c, d)

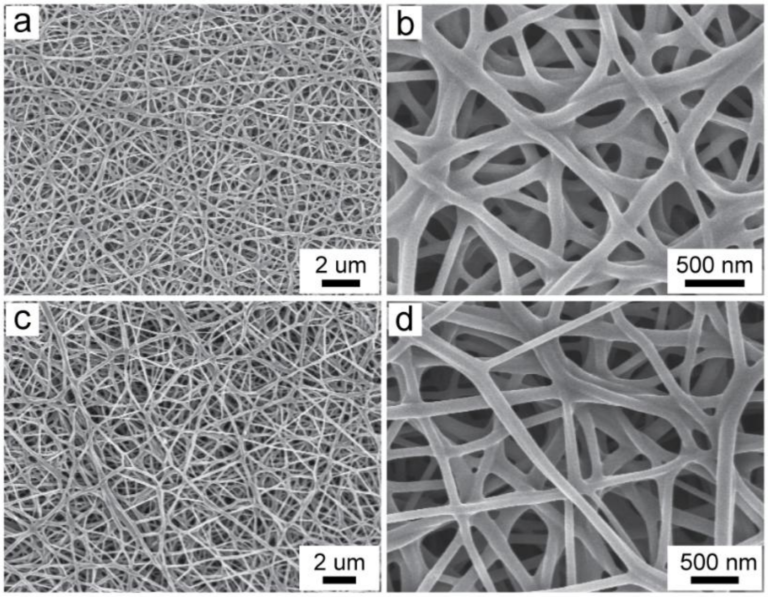

Figure S5. The SEM images of N-CNFs –900 (a, b) and PN-CNFs–900 (c, d)

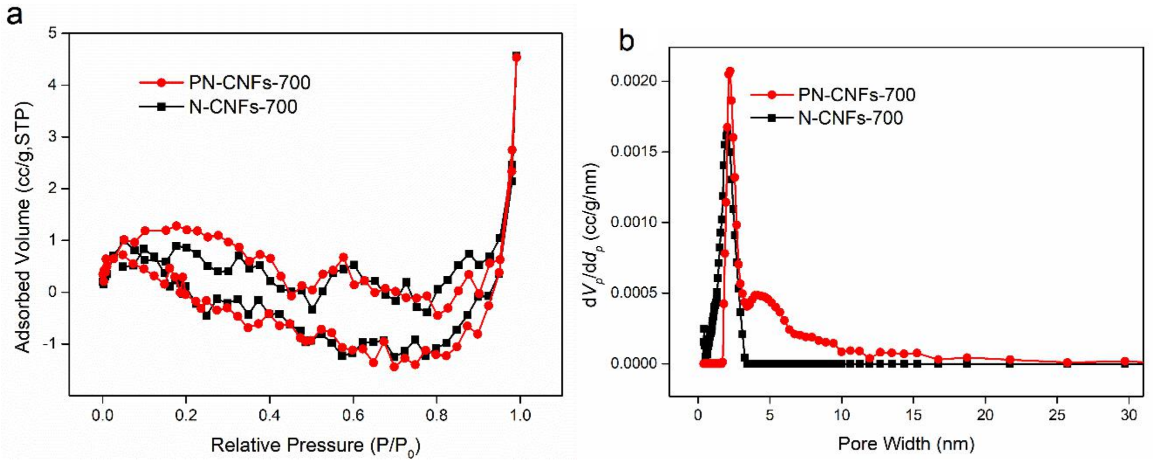

Figure S6. (a) Nitrogen adsorption-desorption isotherms and (b) the pore size distribution of PN-CFs and N-CFs.

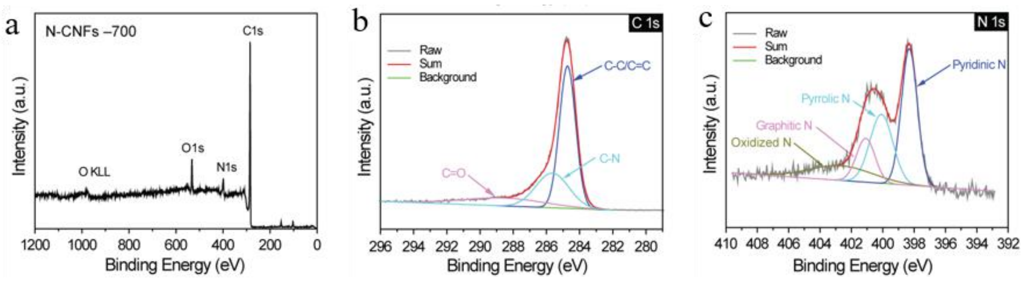

Figure S7. XPS survey (a) and high resolution C1s (b) and N1s (c) spectra of N-CFs-700.

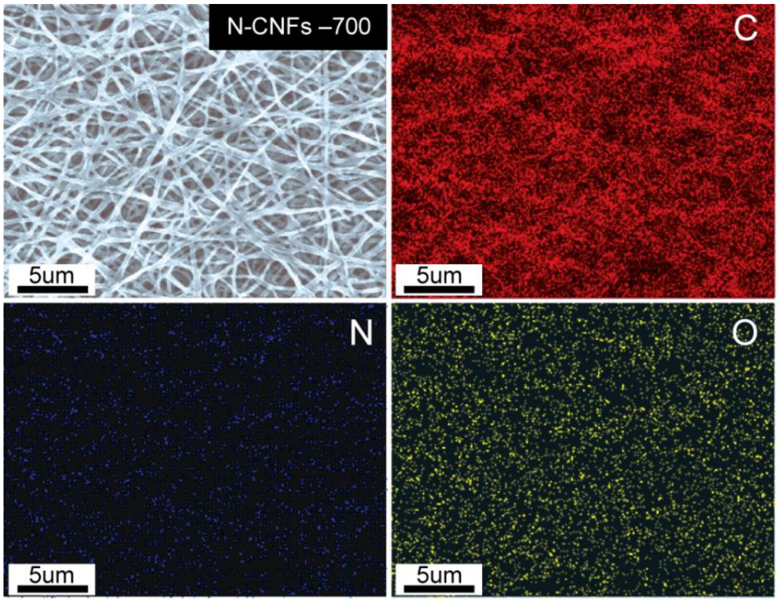

Figure S8. The SEM image and EDS mapping of N-CFs-700

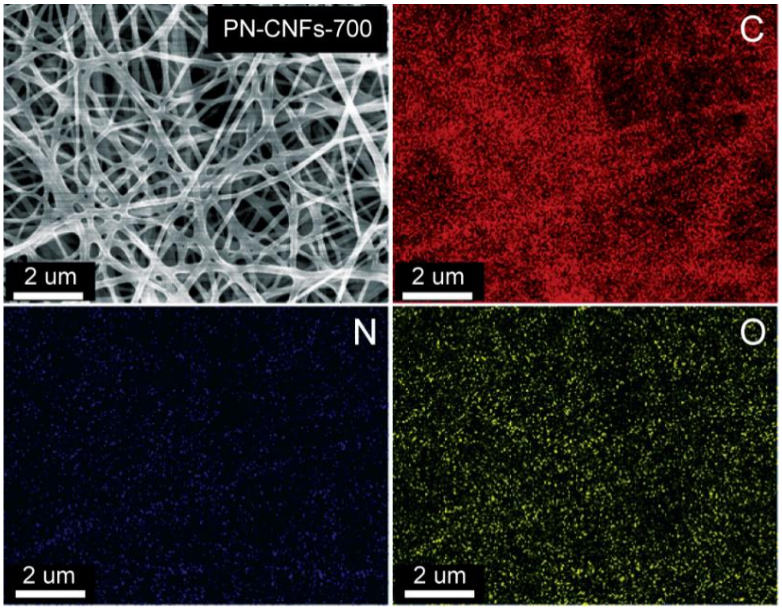

Figure S9. The SEM image and EDS mapping of PN-CFs-700.

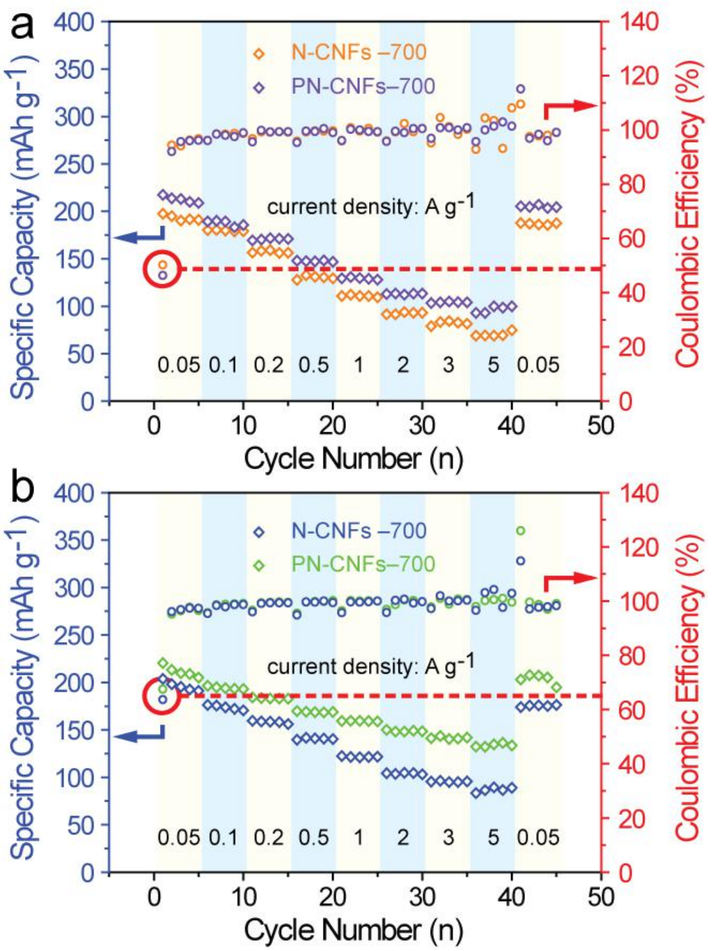

Figure S10. The rate performance of N-CNFs –700 and PN-CNFs–700 with of carbonates (a, EC/PC/FEC, 1 M sodium perchlorate) and ethers (b, diethylene glycol dimethyl ether, 1M trifluoromethyl sulfonate) electrolytes.

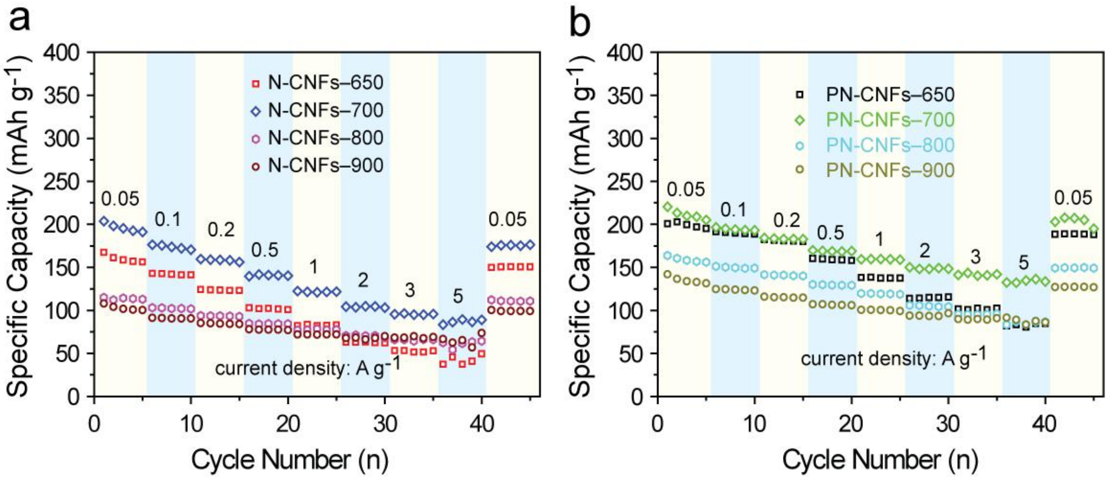

Figure S11. The rate performance of N-CNFs-T (a) and PN-CNFs-T (b) obtained at different carbonization temperatures from 650 °C to 900 °C.

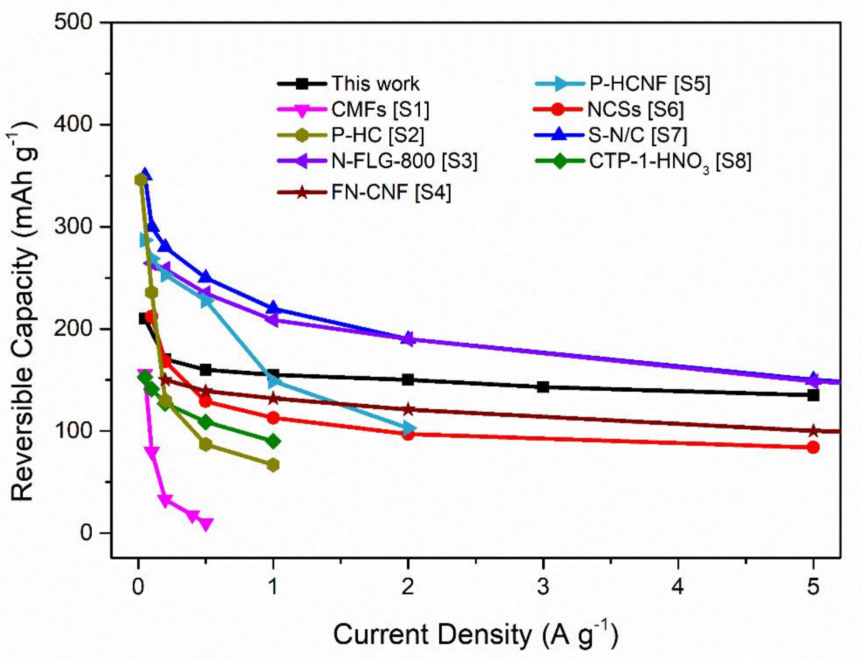

Figure S12. Comparison of the rate performances of PN-CFs fibers with reported carbon materials for SIBs.

References

[S1] Flores, D., Villarreal, J., Lopez, J., and Alcoutlabi, M. (2018). Production of carbon fibers through Forcespinning® for use as anode materials in sodium ion batteries. *Mater Sci Eng B* 236-237, 70-75. doi: 10.1016/j.mseb.2018.11.009.

[S2] Li, Z., Ma, L., Surta, W., Bommier, C., Jian, Z., Xing, Z., et al. (2016). High Capacity of Hard Carbon Anode in Na-Ion Batteries Unlocked by POx Doping. *ACS Energy Lett.* 1. doi: 10.1021/acsenergylett.6b00172.

[S3] Liu, J., Zhang, Y., Zhang, L., Xie, F., Vasileff, A., and Qiao, S. Z. (2019). Graphitic Carbon Nitride (g-C_3N_4)-Derived N-Rich Graphene with Tuneable Interlayer Distance as a High-Rate Anode for Sodium-Ion Batteries. *Adv. Mater.* 31, 1901261. doi: 10.1002/adma.201901261.

[S4] Wang, Z., Qie, L., Yuan, L., Zhang, X., Hu, X., and Huang, Y. (2013). Functionalized N-doped interconnected carbon nanofibers as an anode material for sodium-ion storage with excellent performance. *Carbon* 55, 328-334. doi: 10.1016/j.carbon.2012.12.072.

[S5] Wu, F., Dong, R., Bai, Y., Li, Y., Chen, G., Wang, Z. et al. (2018). Phosphorus-Doped Hard Carbon Nanofibers Prepared by Electrospinning as an Anode in Sodium-Ion Batteries. *ACS Appl. Mater. Interfaces* 10, 21335-21342. doi: 10.1021/acsami.8b05618.

[S6] Yang, F., Zhang, Z., Du, K., Zhao, X., Chen, W., Lai, Y., et al. (2015). Dopamine derived nitrogen-doped carbon sheets as anode materials for high-performance sodium ion batteries. *Carbon* 91, 88-95. doi: 10.1016/j.carbon.2015.04.049.

[S7] Yang, J., Zhou, X., Wu, D., Zhao, X., and Zhou, Z. (2017). S-Doped N-Rich Carbon Nanosheets with Expanded Interlayer Distance as Anode Materials for Sodium-Ion Batteries. *Adv. Mater.* 29, 1604108. doi: 10.1002/adma.201604108.

[S8] Zhuang, Z., Cui, Y., Zhu, H., Shi, Y., and Zhuang, Q. (2018). Coal-Based Amorphous Carbon as Economical Anode Material for Sodium-Ion Battery. *J. Electrochem. Soc.* 165, A2225-A2232. doi: 10.1149/2.0981810jes.
